# Building Resilience Through Sport in Young People With Adverse Childhood Experiences

**DOI:** 10.3389/fspor.2021.663587

**Published:** 2021-07-15

**Authors:** Gareth Norris, Heather Norris

**Affiliations:** Department of Psychology, Faculty of Earth and Life Sciences, Aberystwyth University, Aberystwyth, United Kingdom

**Keywords:** ACEs, well-being, mental health, sport, resilience (psychological)

## Abstract

Interventions focused on young people at risk of anti-social and criminal behaviour frequently involve physical exercise and/or participation in sporting activities as a primary vehicle to bring about behavioural change in both the short to medium term. Anecdotal evidence suggests that sporting activities positively influence individual well-being alongside a sense of purpose and belonging centred around sporting clubs and activities. Empirically, participation in sport has been identified as a key resilience factor for young people with numerous Adverse Childhood Experiences (ACEs) and investment and policy initiatives target investment in these opportunities. However, the psychological mechanisms which underpin these improvements in well-being and building of resilience are less clearly articulated. This article will review current and developing research in this field to synthesise future applications of sport-related interventions with young people, with a particular emphasis on furthering understanding of the pathways to capacity building at individual and community level which sport and exercise can generate.

## Introduction

Adverse Childhood Experiences (ACEs) are a robust indicator of later life health problems, including mental/physical health, substance misuse, criminality and early death (Felitti et al., 1998). However, although the link between ACEs and poor outcomes is fairly well-established, recent research is exploring the effect of identifying protective factors and building resilience in order to mitigate their impact (Ungar, 2013; Bellis et al., 2018). For example, non-substance users with ACEs were significantly less likely to be involved in criminal behaviour than substance users (Craig et al., 2017). Many public health initiatives, such as the Welsh Government's Future Generations and WFG Act (2015), have initiated policy guidance and interventions specifically to build resilience to ACEs. In particular, sporting activity—alongside having a trusted adult relationship (see Bellis et al., 2017)—has been identified as key resilience factor in relation to reducing instances of deterioration amongst people with existing mental illness (Hughes et al., 2018):

*There are strong relationships between sports participation in childhood and lower lifetime mental illness. There are also associations between regular adult participation in sports and current mental illness. While much attention has been paid to the cardiovascular and weight reduction potential of sports participation, its impact of friendship opportunities, benefits to mental health, access to role models and the other aspects of resilience that engagement in sports facilitates needs to be factored into its benefits and further understood (Hughes et al.*, *2018**; p. 8)*.

Although ACEs have been consistently shown to predict several negative life outcomes—including criminal behaviour—it appears that a number of factors can shape and build resilience and mitigate some of the impact (Bellis et al., 2017; Ross et al., 2020). Public Health Wales has been a major source of research into ACEs and resilience. Focusing predominantly on mental health, it is found that having four or more ACEs was closely correlated with negative psychological consequences, but this was significantly reduced for children who had a trusted relationship with at least one adult and also those who regularly participated in sport (Hughes et al., 2018). Alongside these developments, discovering new pathways to building resilience and negating the impact of ACEs are at the forefront of Public Health Wales and Welsh Government's agenda and the WFG Act (2015).

The aim of this article is to attempt to link together many of these concepts (principally ACEs and Resilience) alongside the Future Generations Act, into a broader debate about the role that sport specifically can have on addressing the impact of negative life circumstances. Most importantly, we advocate the development of an evidence-based approach to understanding and evaluating the psychological mechanisms by which sport can serve as a protective factor for ACEs and other areas of detriment in childhood development. We take a critical view of sport as a vehicle for change; simply providing opportunities for sport is unlikely to have a major influence on the mental health of young people on its own. Rather, activities need to be targeted and appropriately resourced, as well as reachable in respects to their location and accessibility; outcomes and processes need to be carefully mapped out and integrated into programme design. Ultimately, we suggest the need for clarity and development in terms of how sporting activities are integrated into the lives of young people—particularly those with evidence of ACEs—and more research into how resilience specifically is both the outcome and the vehicle with which to build improvements in mental health for young people now and into adulthood.

## Resilience, Well-Being, and Mental Health

Building resilience is a key aspect of the ACEs agenda. At a broad level, resilience refers to the way in which people can cope and overcome problems (Bandura, 1995), but more specifically, it relates to thinking patterns that enable positive growth and development in order to remain positive in the face of adversity. The term was largely developed by Rutter (1987) to explain: “[…] developmental and situational mechanisms involved in the protective processes” (p. 2). In essence, this was more than examining protective factors and the study of resilience shifted emphasis to focus on how people *overcame* emotional, developmental and economic challenges in childhood and beyond. With increasingly complex situations being faced by young people in the ever-changing modern world, the study of resilience has become an important way to understand how young people make the successful transition into adulthood, particularly when they have encountered severe difficulty and hardship along the way, for example, experiences with mental health (see Ungar, 2019) and/or more generalised adversity (see Höltge et al., 2021). The development of a resilient mind-set has become the focus of much recent research; many longitudinal studies suggest that this is something which can be developed in all children and to a lesser degree taught as a coping mechanism for a range of adverse experiences, for example, survivors of natural disasters (see Kingston et al., 2019). Recent research exploring resilience and the Covid pandemic (see Killgore et al., 2020), has shown that exercise featured amongst a range of factors that predicted higher levels of reported resilience.

One specific aspect of resilience relates to well-being and the “healthy individual” that can interact within specific environments (see Cowen, 1991). Happiness (or subjective well-being) is a generalised term used to describe the affective and cognitive evaluations of one's life (Diener, 2000). There is a newly found importance being placed on these measures following the results of large-scale international comparisons and subsequent government legislation (for example, the Welsh Government Social Services and Well-being Act 2016, Wales). Importantly, Holder and Coleman (2015) suggest that factors such as income are largely unrelated to overall happiness and generalised well-being, even when people are aware of their marginalised standings economically. International research into well-being has demonstrated that happiness levels are high in some economically disadvantaged countries and lower in more prosperous ones—a process known as satiation—where increasing wealth does not add significant value to life satisfaction (see Jebb et al., 2018). Subsequently, there are several factors that contribute to overall happiness in young people; one key finding is that demographic elements contribute to a relatively minor proportion of children's levels of well-being (Gilman and Huebner, 2003). A larger body of research has examined generalised intelligence specifically (and subsequent educational ability) and found that the direct impact is largely unrelated to happiness levels in children (see Huebner and Alderman, 1993). Additional factors such as the interaction between emotional intelligence and stress (Ruiz-Aranda et al., 2014) and self-esteem (Sillick, 2006) have demonstrated the mediating individual differences that influence the ability to remain positive in the face of adversity. Some of these additional elements are areas that schools have the ability to influence, such as building positive relationships and increasing student participation in both the educational syllabus and extra-curricular activities including sports (Lyubomirsky et al., 2005; Huebner and Diener, 2008). In particular, Ungar (2017) have shown that schools may have the greatest impact on resilience among children who are the most disadvantaged.

## Adverse Childhood Experiences (ACEs)

Theoretically and empirically, ACEs can be regarded as a range of risk factors linked to traumatic events that have been identified as useful in predicting a range of negative health and social outcomes (Ross et al., 2020). From 1995 to 1997, Kaiser Permanente's Health Appraisal Clinic, in collaboration with the Centre for Disease Control and Prevention (CDC), conducted the largest ever study on the origins of risk factors associated with negative health. The results confirmed that the more negative experiences in childhood the greater health, behavioural and social impact in adulthood (CDC, 2014). The prevalence of ACEs in the original study was somewhat unexpected as the participants in the original study were largely drawn from middle/upper middle class white college educated population; generally speaking, a cohort with expected access to quality health-care services. The majority of the sample, nearly two-thirds, had at least one ACE, and of these, a further 87% had two or more ACEs. The extent of potential negative health outcomes includes conditions such as lung cancer and early death and subsequent reductions of life expectancy of up to 20 years for those with the highest number of ACEs (Hughes K. et al., 2020). The original core 10 ACEs first identified by the (CDC) relate to: physical abuse, sexual abuse, verbal abuse, physical neglect, emotional neglect, a family member who is depressed or diagnosed with other mental illness, a family member who is addicted to alcohol or another substance, a family member who is in prison, witnessing a parent/family member being abused, and losing a parent to separation, divorce or death (Felitti et al., 1998). Whilst originally linked to adverse health, the increased likelihood of juvenile offending has also been reliably predicted by ACE evaluations (Wolff et al., 2017; Hughes N. et al., 2020). ACEs are detrimental to later life outcomes, health and well-being, and importantly several indicators have been shown to exert cumulative stress upon an individual and limit brain development in young people (Anda et al., 2010; Guinn et al., 2019). Wolff et al. (2017) recorded a strong correlation between the number of ACEs and general offending; however, those with more ACEs were also likely to be re-arrested and have shorter recidivist periods.

Adverse Childhood Experiences (ACEs) include a range of different types of traumatic experiences a child may be exposed to including both prolonged stressful events or acute occurrences. The original ACEs study operationalised childhood exposures to trauma within seven broad categories: psychological abuse, physical abuse, sexual abuse, substance abuse, mental illness in the household, mother treated violently, and criminal behaviour in the household (Felitti et al., 1998). From this each participant received a composite score from 0 (unexposed) to 7 (exposed to all categories). More recently further categories of ACEs have been incorporated to account for adversity beyond the initial houseful including community and systemic causes such as exposure to racism and chronic poverty. Exposure to Adverse Childhood Experiences contribute to a lasting range of physical, behavioural, emotional, and mental health issues in adulthood, the prevalence of which and the impact on health and social services are just now being understood. After taking socioeconomic and demographic factors into account it is found that four or more ACEs increases the incidence of a range of chronic health issues, including an increased likelihood in experiencing lung disease, heart disease, depression, attempted suicide, anxiety, and disability (Campbell et al., 2016; Ranjbar and Erb, 2019). The medical implications of high ACE scores are profound but additional biological and socio-behavioural are equally overwhelming. There is evidence that high level of ACEs is linked to both chromosomal changes and functional issues with the developing brain (Anda et al., 2006, 2010). Higher ACEs also increase the likelihood for risky sexual behaviour, teenage pregnancy, substance abuse, heavy drinking, violence behaviour, and poor educational and employment outcomes (Anda et al., 2001; Hills and Waldfogel, 2004; Bellis et al., 2014a,b).

Studies have expanded since the focus on the original demographics to include children from a range of socioeconomic backgrounds. Some cohorts, for example young people who have experienced periods in foster care, have particularly high levels of recorded psychological distress (Bruskas and Tessin, 2013) and overall ACEs (Turney and Wildeman, 2017). More recent research has included juveniles in the justice system, where ACE studies repeatedly find that higher rates of childhood trauma within incarcerated populations; Reavis et al. (2013) reported ACE scores four times than that in the general population. The social repercussions on communities and the financial burden on health services also has implications for these children's individual health and well-being throughout the life course. Bellis et al. (2014a) report that non-communitable diseases account for two-thirds of all deaths, with alcohol and tobacco use being the two-leading health-harming behaviours. Such health-harming behaviours are typically more prevalent in those sectors of society with the lowest income and significantly increase with the rise in ACE scores. Bellis et al. (2019) estimated the annual cost attributed to ACEs at US$581 billion in Europe and $748 billion in north America and that three-quarters of the expenditure were on those with two or more ACEs; a 10% reduction in the prevalence of ACES would equate to ~$105 billion saving in ACEs health expenditure in both regions. However, there are systemic issues resulting in a “silo mentality” between agencies and a historical focus on the “disease-model” of care which produces a focus on adult medical care, neglecting the root of the problem (Corso et al., 2008). Seemingly, the evidence suggests that to achieve an overall reduction in ACEs and their associated impact on physical and mental health, agencies must share the responsibility to refocus on childhood prevention and interventions (see Atchison et al., 2020).

The growing body of evidence clearly exposes Adverse Childhood Experiences (ACEs) as having detrimental and enduring consequences impacting on both physical and mental health throughout a lifespan (Ross et al., 2020). Confronting this issue is complex and requires collaborative efforts between a range of agencies, community involvement and the building of resiliency, many of which are not often achievable within current governmental and agency structures (Bethell et al., 2017). However, Wales has enacted new legislation requiring the well-being of its citizens to be at the heart of all decision making and through this regulation it is possible to require collaborative thinking and novel approaches to addressing the impact of ACEs. Through the WFG Act (2015) it is possible to use sport as a mechanism for asset-based approaches to building resilience in both the individual communities. Emerging areas of research, including the salutogenic model of health, which describes how well-being develops in challenging situations and offers an interesting perspective on the mechanisms underlying youth developmental processes (Super et al., 2021).

## The Link Between Sport and Positive Mental Health

One major issue in discussing the impact of sport and exercise generally and the link with mental health and well-being, is being able to understand the complex and often misunderstood relationship between these factors (Donaldson and Ronan, 2006). Confounding the issues in defining the pathways between sport and well-being are the ambiguous terminology and definitions of these constructs themselves. For example, engaging in “sporting” activities can mean different things, both in terms of the type of activity, such as individual, social or team, as well as intensity, duration, and level (Lundqvist, 2011). Similarly, defining well-being and measuring improvements is complicated and mental health problems more generally are prone to fluctuations over time and the lifespan generally (Schönfeld et al., 2017). Resilience also, as discussed, can range in outcomes and our specific understanding of how this manifests itself within the individual and how remains under development (Ungar, 2019). Hence, understanding the impact of sporting activity on physical and mental health necessitates a level of interrogation beyond the basic generic assumptions of weight loss and positivity, etc.

At a basic level, most sporting events are related to increased cardiovascular activity and there are proven links to the positive impacts on physical health, for example, obesity and heart disease (Pastor et al., 2003). Many studies have also demonstrated a similar influence on psychological outcomes, including reducing levels of depression and stress coping in adults (Newman and Motta, 2007). An early review of these studies by Scully et al. (1998) presents a wide range of research in support of the way in which self-esteem and anxiety can be alleviated by exercise. Similarly, Salmon's (2001) critical review, articulates regular exercise as a “controllable stressor” which can essentially accelerate the clinical development of coping mechanisms within the individual. That is, stress is induced and then controlled by the very process of exercise. More recent research focuses on the mechanisms by which emotional regulation is optimised by counteracting the effects of stress upon the individual (Bernstein and McNally, 2018). Stressful incidents, whether linked to post-traumatic disorders (see Lawrence and Kisely, 2010, for a review), or longer-term events such as ACEs, identify how sporting activity has the ability to demonstrate empirically supported gains:

“*Regular childhood sports participation was associated with lower levels of mental illness across all ACE levels. Among those with four or more ACEs who regularly participated in sports in childhood, the adjusted proportion reporting: Lifetime mental illness was 49%, compared with 55% in those not participating in sports; Current mental illness was 19%, compared with 25% in those not participating in sports. Having ever felt suicidal or self-harmed as 25%, compared with 34% in those not participating in sports” (Hughes et al.*, *2018**; p. 11)*.

Despite the overwhelming evidence of the psychological and well-being benefits associated with sports for young and old, governments and policy makers have notoriously found these positions difficult to achieve (Hall and Reis, 2019). However, a minority of countries have added legislation ensuring the well-being of its citizens are at the heart of all governmental and public body decision making, capturing a range of well-being indices, which goes beyond the national GDP as a reflection of broader aspects of living conditions and quality of life. Alongside Wales' WFG Act (2015), implemented in April 2016, other examples include New Zealand's well-being budget (see Anderson and Mossialos, 2019), which addresses the need to provide resources to boost the mental and physical health of its citizens.

WFG Act (2015) put into place seven core well-being goals, making it a legal obligation that public bodies consider each goal in all its decision-making practises. The goals include: A prosperous Wales, A resilient Wales, A more equal Wales, A healthier Wales, A Wales of cohesive communities, A Wales of vibrant culture, and A globally responsible Wales. To achieve these goals the underlying principle of sustainable development remains at the core as a driver to ensure all decisions are made with the well-being of future generations of Wales in mind. Public bodies must make present decisions without compromising future generations good quality of life but also consider how to stop problems arising initially (Welsh Government, 2016).

To ensure that public bodies do not fail to achieve its intended well-being goals The Well-being of Future Generations Act requires all agencies to demonstrate their legal obligations of sustainability through five “ways of working” (Future Generations Commissioner for Wales, 2020):

1. Long term: The importance of balancing short-term needs with the needs to safeguard the ability to also meet long-term needs.
2. Prevention: How acting to prevent problems occurring or getting worse may help public bodies meet their objectives.
3. Integration: Considering how the public body's well-being objectives may impact upon each of the well-being goals, on their objectives, or on the objectives of other public bodies.
4. Collaboration: Acting in collaboration with any other person (or different parts of the body itself) that could help the body to meet its well-being objectives.
5. Involvement: The importance of involving people with an interest in achieving the well-being goals, and ensuring that those people reflect the diversity of the area which the body serves.

These objectives feed down into the local level via the Public Services Boards^1^, compromising a diverse group of stakeholders of both statutory (Chair of the council, health board, Natural Resource Wales, Fire and Rescue Service) and invited partners (e.g., representatives from local universities, registered social landlords, and police and crime commissioner) from the local authority. The locally led Public Services Board incorporates the national agenda but tailors the overall well-being goals to their resident location, looking at the needs of the regional population (WFG Act, 2015, Part 4 “Public Services Boards” section 29). Through the sustainability principles separate agencies, who usually do not work together are able to draw on their own resources to fulfil their legal obligations of long-term needs, prevention, integration, collaboration, and involvement. The legislation precludes the “silo-mentality” which afflicts other larger nations and their ability to improve well-being as mentioned by Bellis et al. (2019). The practical applications of the sustainability principle have the ability to break-cycles and recognise the cause and effect of different issues that the local communities face by assessing and planning in a more holistic manner compared to the traditional working model. Similar to national public bodies, the Public Service Board must publish annual plans documenting how they will meet the needs of future generations under the remit of the WFG Act (2015).

Many Public Services Boards have applied the sustainability principle to create novel practises best fit for regional variations of need. By doing so allows for a move away from the conventional disease-model of health and care and a move toward an assets-based approach to intervention in the community. An asset-based approach is considered a “strengths based” framework where all individuals and communities are assumed to their own capacities to improve or change their lives (Zeeman et al., 2016)^2^. Such an approach places an emphasis on social connectedness, leaving individuals and communities with a sense of control over their lives, and ultimately improving resiliency in the face of adversity (Foot, 2012). Salutogenic models (see Super et al., 2018), have demonstrated the potential for community sports to have a positive impact on individuals through the instillation of small personal successes and fostering a sense of belonging to the group.

## Asset-Based Approaches, ACEs, and Resilience

Although health outcomes are generally improving, the gap between the most affluent and the highest deprived are increasing. Asset-based approaches are one method to decrease these inequalities between communities where established protocols have failed and works by highlighting what strengths and capabilities both the individual and community fosters, and how using these can lead to lasting behavioural change (Foot and Hopkins, 2010; Misener and Schulenkorf, 2015). The Future Generations report comments on both achievements and deficiencies in specific local authorities prioritising opportunities through sports, for example, Vale of Glanmorgan council was commended as a model example for community engagement and articularly significant is its intergenerational work and the incorporations of community volunteers to drive its community activities. In this way, the local community can modify and increase existing resources thus expanding community connexions and building resilience. Capitalising on individual and community strengths through an Asset-based lens has potential to influence on health promotion strategies, and sport is one such avenue which can help to release this potential (Whiting et al., 2012; Super et al., 2018).

There does, however, need to be a mechanism by which the various elements of any asset-based approach can develop and resilience is one concept that has become more prominent in contemporary discourse surrounding positive mental and physical health (Höltge et al., 2021). Resilience is often defined as the ability to “bounce back” after a traumatic or stressful event (Guimarães, 2018) and the literature on the importance of resilience has increased dramatically in recent years possibly due to its malleable nature and its ability to influence life outcomes even in the face of adversity (Ungar, 2017). Although the definition of resilience is understood as an individual protective factor built from personal strengths to overcome adversity, there is a growing consensus on the role of supported and connective social contexts into the understanding and development of the concept (Panter-Brick and Leckman, 2013). Social connectiveness can have a significant impact on the building of resiliency. However, the most deprived areas largely report a general lack of social support, leading to less resiliency and increased likelihood in engagement with unhealthy behaviours. An asset- based approach can build community-based capabilities and promote resilience through interventions as it shifts the focus from the disease and deficit models of health to a capacity and strengths based (Foot, 2012). The importance of asset-based intervention to those most at risk of ACEs is important; generally, as the number of ACEs increase, reliance resources decrease. For those adults with four or more ACEs in Wales, it was found that some reliance resources reduced the negative mental health outcomes by more than half (measured using a scale including relationship, personal and community resilience factors) (Hughes et al., 2018). The provision of sports-based activities has been shown to have a direct influence on the way in which areas with high levels of deprivation in particular, can benefit from community-level interventions (Agnew, 2013).

Sports and physical activity are associated with better physical and mental health, and reduced risk of health-harming behaviours (Steiner et al., 2000; Malm et al., 2019). There are also a host of societal benefits including increased pro-social behaviour, reduction in crime and antisocial behaviour, and improved social connectedness (Chamberlain, 2013). Despite the wealth of research on the physical, mental, and social impact of sport participation and the wider social value to participation since the inception of austerity measures starting in 2008, sports across nations have endured funding cuts and reduced investment to more immediate health interventions in the UK. For example, Widdop et al. (2017) found that since 2008, English local authority expenditure reductions on sport resulted in not meeting any goals for widening participation for “hard to reach groups.” However, other commentators recognise the importance of developing new model of sports participation based on the assets-approach that are immune to the severe economic constraints still currently felt by many nations which spans generations, not just the young:

“*Access to sources of resilience in adulthood continues to be associated with lower levels of current mental illness. Along with sports, positive relationships were found with engagement in community and social groups, enjoying community culture and traditions, longer perceived financial security, and higher perceived support from public services and employers. Focus should include developing opportunities for individuals to increase their resilience resources across the life course, to offer protection from the adverse effects of ACEs as well as trauma that may occur in adulthood” (Hughes et al.*, *2018**; p. 10)*.

Generally, sports development relies on funds for equipment, space and human resources. The issue with this model is that it relies on the continuation of funds to maintain the sporting activity. Since the economic crisis of 2008, sporting facilities have either had to reduce opening hours or close completely, additionally the price of admission and the cost of participation has increased, ultimately impacting the level of participation for those in deprived areas. Rossi and Jeanes (2018) support the notion of a maintainable capabilities approach, where rather than continue the dependency on the conditions of the global markets for economic support, communities must develop a sustainable framework based on the local assets, both human and physical, for sports programmes.

Wales is at the forefront of sports policy in relation to widening participant and asset-based approaches. Wales' sport policy delivered primarily through Sport Wales, a national organisation responsible for developing and promoting sport, acknowledges the need for community involvement and has a range of funding opportunities to help develop sustainable community programmes. Sports Wales research found that investments of £1 in sports return of £2.88. They report nearly a £3.5 million benefit from participating and community volunteering in a sporting activity. The value of improved health was estimated at £295 million and the benefit to the social capital in Wales rose to £651 million, where 60% of this value is enhanced subjective well-being. Overall, Wales demonstrates an economic return on investing in sustainable sports and physical activity programmes for the national well-being of the nation.

“*In addition to overall adult resilience, lower levels of current mental illness were associated with regular sports participation; regular participation in social clubs/community groups” (Hughes et al.*, *2018**; p. 8)*.

Sports participation has long been known as a factor for resilience building but more recently research has focused on the importance of sport as a moderating factor for those with ACEs (White and Bennie, 2015). Welsh policy makers and public bodies are cognisant of the direct positive impact of sport on the prevention and intervention of ACEs through a number of recent publications by Public Health Wales. Public Health Network Cymru and Public Health Wales both equally support the importance of sport and widening participation in regard to ACEs. Public Health Wales reports that “of childhood activities measured, only regular participation in sports showed a protective effect against mental illness. Among those with four or more ACEs, the adjusted proportion reporting current mental illness fell from 25% of those who did not regularly participate in childhood sports to 19% in those who did” (Hughes et al., 2018, p. 35). Furthermore, the Chief Executive of Public Health Wales states that building resilience in both child and adulthood is a key intervention needed to overcome issues arising from ACEs (Hughes et al., 2018, p. 1). The ambition and evidence is clearly developed; however, there is an urgent need to understand how the investment will deliver the change required in order to be justified by generating impact upon key health and social indices such as well-being, obesity, crime rates, amongst others.

## Resilience and the Global Pandemic

Writing this in the middle of a global pandemic means that thoughts must extend to understand what the impact of Covid-19 (and potential future outbreaks) may mean for young people. Clearly, the curtailment of sports clubs and venues will mean that young people have limited outlet for engagement in this vital activity has been limited and in turn may further impact on the way in which mental health pressures associated with the pandemic have been exasperated:

“*Poor mental health may reduce opportunities for community engagement, while lower engagement may further impact on mental ill health. Specific interventions may be essential to breaking this pathological cycle, especially where it is well-embedded. However, developing community resilience resources and supporting those with high ACEs and low resilience at the earliest possible stages should offer a more effective mechanism to improve population well-being” (Hughes et al.*, *2018**; p. 12)*.

Whilst cycles of pathology are already endemic amongst many deprived communities; the pandemic has highlighted also the extent of these disparities, particularly BME populations, and intervening in the added pressures from the pandemic is vital. The evidence from major natural disasters (see Mcdermott et al., 2010), suggests that families with low resilience and previous exposure to mental health problems are likely to suffer disproportionately to these unfavourable environments.

But the pandemic does create an opportunity to re-establish sporting opportunities for young people in a meaningful and directed way. We are likely to come out of this pandemic with a generation of young people who have low levels of well-being and physical and mental health, and potentially increased exposure to ACEs. To successfully address the problems likely to emerge from this, the design and implementation of successful sports programmes that engage young people and build resilience specifically are of paramount importance. Creating opportunities to re-start sports programmes will enable greater attention to be placed on engagement and identifying the ways that exercise and the wider benefits of sports can alleviate the negative impacts on young people's mental health. The challenge that remains is unpacking the link between resilience as a psychological construct and how this translates into a protective personal growth skill that can be nurtured through sport. One thing that the pandemic has illustrated is the basic human need for exercise and social contact, which sport can facilitate and cultivate.

## Conclusions and Future Directions

It is clear that sport and physical activity have the potential to influence both physical and mental health; related to this is the notion that these benefits can be translated into factors associated with resilience and therefore serve as a protective factor against many negative life events, including ACEs. However, research in this area has distinctly lacked either the empirical support and/or has focused on shorter-term impacts, which largely fail to fully articulate the psychological processes by which these “changes” are enacted and maintained within the individual. Many accounts are anecdotal; more empirical research and specifically longitudinal data is needed to better identify these processes:

“*It should be noted that results from this survey alone cannot establish cause and effect between resilience resources and mental health. We recognise that causality is likely to run in both directions and are interested in exploring the opportunities for resilience to prevent mental illness. Work needs to be undertaken to identify which interventions can work to build resilience in Wales and to ensure that effective programmes are implemented” (Hughes et al.*, *2018**; p. 14)*.

Research into ACEs, resilience and sport—although well-established in their own respective domains—are really only in their infancy as an approach to addressing well-being deficits. The Future Generations Act in Wales is one of the first pieces of formal legislation which defines a commitment to provide young people with the support and skills necessary to contribute to their current and future well-being. We advocate that this approach is a vital part of the Future Generations agenda, whether that be in counteracting the negative influence of ACEs on life-outcomes through building resilience, improving mental health generally through developing and maintaining healthy lifestyles, creating community and social opportunities and a sense of belonging which can continue into later adulthood and also to negate the as yet to be assessed impact of the Covid-19 pandemic on physical and mental health.

## Author Contributions

Both authors listed have made a substantial, direct and intellectual contribution to the work, and approved it for publication.

## Conflict of Interest

The authors declare that the research was conducted in the absence of any commercial or financial relationships that could be construed as a potential conflict of interest.

## Footnotes

^1^https://gov.wales/sites/default/files/publications/2019-02/spsf-3-collective-role-public-services-boards.pdf

^2^Nice (2020). https://static.nice.org.uk/NICE%20strategy%202021%20to%202026%20-%20Dynamic,%20Collaborative,%20Excellent.pdf
